# Psychological Laboratories

**Published:** 1888-09

**Authors:** 


### PSYCHOLOGICAL LABORATORIES.

The study of psychology as an experimental science is likely to advance, considering that laboratories for research have been, or are now being, established in many of the Continental towns and in America. Among these we may mention Leipzig, Berlin, Bonn, and Göttingen ; in America, at Johns Hopkins, Harvard, Pennsylvania, and Princeton; while at Cambridge we have the anthropological laboratory which Mr. Francis Galton has done so much to establish. Many phenomena concerning mental action are capable of being reproduced for the purposes of accurate study and comparison with the hope of attaining exact knowledge. Some of the most valuable results have been obtained in studying simple phenomena, as seen in children ; but experiment is wanted as a means of keeping certain conditions constant, and for obtaining records of phenomena capable of objective examination and measurement. Dr. James McKeen Cattell, of the University of Pennsylvania, has given an account of the work done in the psychological laboratory of Leipzig in *Mind*, January, 1888.

Experiments have been made as to the measurement of sensation, both by varying the pressure upon parts and by determining the variations in illumination that can be appreciated by the eye. Sound and light have both been used in investigations as to the relations between stimulus and sensation. Many researches have been undertaken to determine the duration of mental processes; there is no doubt that mental processes take up time, and that this time may be measured. It would be very interesting to learn whether, in the course of evolution in man, the physical processes which correspond to thinking have become shorter or longer, or have remained unchanged. There is no doubt that the interval between stimulus and response varies much in different persons. Dr. McKeen Cattell concludes by saying: "The results obtained in the Leipzig laboratory during the past seven years have been briefly noticed. They prove conclusively that it is possible to apply experimental methods to the study of mind. The positive results are, besides, not insignificant, and will compare favorably with what has been accomplished during the same period in many chemical, physical, and physiological laboratories. An increased interest is everywhere being taken in experimental psychology, and we may hope that we shall some day have as accurate and complete knowledge of mind as of the physical world." It is to be hoped that, in the arrangement of the Royal Colleges for providing laboratories in London, place will be found for the encouragement of research in the departments of physiological psychology and anthropometry, and that in a few years the reputation of the work in this department may be such as may partially remove the indebtedness of England to other countries. —*The British Medical Journal.*

